# A New Non-stationary High-order Spatial Sequential Simulation Method

**DOI:** 10.1007/s11004-022-10004-2

**Published:** 2022-06-16

**Authors:** Amir Abbas Haji Abolhassani, Roussos Dimitrakopoulos, Frank P. Ferrie, Lingqing Yao

**Affiliations:** 1 COSMO – Stochastic Mine Planning Laboratory, McGill University, Montreal, QC Canada; 2 Centre for Intelligent Machines, McGill University, Montreal, QC Canada

**Keywords:** High-order spatial statistics, Sequential simulation, Non-stationary, Transformation-invariant and multi-point statistics

## Abstract

A new non-stationary, high-order sequential simulation method is presented herein, aiming to accommodate complex curvilinear patterns when modelling non-Gaussian, spatially distributed and variant attributes of natural phenomena. The proposed approach employs spatial templates, training images and a set of sample data. At each step of a multi-grid approach, a template consisting of several data points and a simulation node located in the center of the grid is selected. To account for the non-stationarity exhibited in the samples, the data events decided by the conditioning data are utilized to calibrate the importance of the related replicates. Sliding the template over the training image generates a set of training patterns, and for each pattern a weight is calculated. The weight value of each training pattern is determined by a similarity measure defined herein, which is calculated between the data event of the training pattern and that of the simulation pattern. This results in a non-stationary spatial distribution of the weight values for the training patterns. The proposed new similarity measure is constructed from the high-order statistics of data events from the available data set, when compared to their corresponding training patterns. In addition, this new high-order statistics measure allows for the effective detection of similar patterns in different orientations, as these high-order statistics conform to the commutativity property. The proposed method is robust against the addition of more training images due to its non-stationary aspect; it only uses replicates from the pattern database with the most similar local high-order statistics to simulate each node. Examples demonstrate the key aspects of the method.

## Introduction

The uncertainty of a spatially distributed and varying geological attribute can be quantified by analyzing the variation in this attribute within a set of geostatistical or stochastic simulations. Second-order spatial simulation methods (Journel and Huijbregts 1978; David 1988; Goovaerts 1997; Chiles and Delfiner 2012) have been used to quantify spatial uncertainty. Since the early 1990s, a new multiple-point statistics (MPS) spatial simulation framework has been developed to overcome the limitations of previous approaches (Guardiano and Srivastava 1993; Gómez-Hernández and Srivastava 2021; Strebelle 2002, 2021; Journel 2005; Liu et al. 2006; Arpat and Caers 2007; Hu and Chugunova 2008; de Vries et al., 2008; Mariethoz et al. 2010; Honarkhah and Caers 2010; De Iaco and Maggio 2011; Stien and Kolbjørnsen 2011; Chatterjee et al. 2012; Lochbühler et al. 2013; Rezaee et al. 2013; Toftaker and Tjelmeland 2013; Mustapha et al. 2014; Strebelle and Cavelius 2014; Zhang et al. 2017). MPS methods are able to capture and produce more complex spatial patterns than second-order spatial simulation methods by using multiple-point spatial templates that replace the well-established two-point spatial statistics. In the MPS framework, sample data are used as the conditioning data, while the multiple-point spatial relations of the simulations are captured from patterns extracted from a training image (TI) or analogue. As a result, MPS simulations exhibit the statistics of the TI instead of the data, which raises the issue of statistical conflicts between the samples and the TI in circumstances in which sample data are relatively abundant, such as in mining applications (Osterholt and Dimitrakopoulos 2007; Goodfellow et al. 2012). High-order spatial simulation methods (HOSIM) have been developed to address this issue and to provide a new geostatistical simulation framework that deals with complex spatial patterns. HOSIM does not require distributional assumptions and is mathematically consistent (Dimitrakopoulos et al. 2010; Mustapha and Dimitrakopoulos 2010a,b, 2011a; Mustapha et al. 2011, 2014; Tamayo-Mas et al. 2016; Minniakhmetov and Dimitrakopoulos 2016; Minniakhmetov et al. 2018; Yao et al. 2018). It should be noted that in HOSIM methods, the conditional probability distribution function (CPDF) is modelled by a set of orthogonal polynomials, Legendre polynomials in particular (Abramowitz 1974). Under the stationarity assumption, high-order spatial cumulants (Dimitrakopoulos et al. 2010) are inferred by averaging the same-order cumulants of the samples extracted from either the data set alone, if sufficient data are provided, or both data and TI in the case of sparse data. The high-order statistics enable the CPDF model to capture the complex spatial structures and connectivity of the high values within the data and to later reproduce them via the simulation process (Mustapha and Dimitrakopoulos 2011a, b; Minniakhmetov et al. 2018; de Carvalho et al. 2019). Stationarity is a major assumption made by the commonly employed spatial simulation methods and may not always be easily accommodated in applications. The traditional approach assumes that a mean function (drift) exists as a linear or polynomial trend. The so-called universal kriging (David 1977) considering the drift, however, may be difficult to apply to some cases (Cressie 1986). More recent research adopts the local probability distribution to model the non-stationary distribution, but only second-order statistics are considered (Machuca-Mory and Deutsch 2013). With respect to high-order simulations, the same assumption also results in the use of all patterns extracted from data and the TI for the estimation of spatial cumulants, leading to the slow convergence of the orthogonal polynomials used for modelling CPDFs. In practice, this calculation may result in numerical instabilities (Boyd and Ong 2011). Thus, a non-stationary approach is used herein to address these issues. For each simulation, a set of patterns are chosen from the data and TI based on their similarities to the data event of the simulation pattern. Then, only the chosen patterns are used for inferring the high-order statistics of the simulation pattern. Similar patterns could be generated from a simple CPDF model with faster convergence using a lower number of polynomial terms.

The high-order sequential simulation method introduced here first finds a data event of a fixed size $n$ in the immediate neighbourhood of each simulation node, and a set of $n+1$ high-order spatial statistics is then calculated from the data event. A novel similarity measure is then introduced and utilized to compare the extracted statistics from the simulation grid to those extracted from all of the patterns in the TI; the statistics with the highest similarity score are then used for the inference of the high-order statistics at that node. This is the non-stationary aspect of the method, which is the key difference between this method and previous high-order simulation methods (Mustapha and Dimitrakopoulos 2010b, 2011b; Minniakhmetov et al. 2018, Minniakhmetov and Dimitrakopoulos 2021; Yao et al. 2018, 2020, 2021). The proposed method avoids an excessive number of patterns extracted from the TI due to the selectivity of the method in terms of choosing the number of related patterns. In addition, the method is able to efficiently identify patterns with similar statistics in different orientations without reducing the computational efficiency of the algorithm.

In the following sections, the proposed method and corresponding algorithm are first detailed. Then, examples and comparisons are presented, and conclusions follow.

## The Method

### Spatial Random Field

Given a probability space $(\Omega ,\mathcal{F},P)$, a function $Z\left(X\right):{\mathcal{D}\subset R}^{\mathcal{N}}\to R$ is a real-valued spatial random field given that for any integer number $n\ge 0$ and $\forall r\in {R}^{n+1},$ a subset ${A}_{r}=\{Z(\mathcal{X})\le r\}\in \mathcal{F}$ exists and its probability is defined by $P$, where $\mathcal{D}$ is the spatial domain in a two- or three-dimensional space ${R}^{\mathcal{N}}(\mathcal{N}=\mathrm{2,3})$ and $\mathcal{X}\subset \mathcal{D}$ is a set of $n+1$ points in this domain. Note that $\mathcal{X}=\{x,{x}_{1},\dots ,{x}_{n}\}$ and $Z(X)=\{Z(x),Z({x}_{1}),\dots ,Z({x}_{n})\}$ and thus the binary operation $\le$ in ${A}_{r}=\{Z(\mathcal{X})\le r\}$ is true only if all elements on the left-hand side are less than or equal to the elements on the right hand side of the equation.

### Template, Pattern, Data Event and Neighbourhood

Consider a random field $Z\left(X\right)$ with a fixed structure in space and a spatial template $T$ that is characterized by a set of lag vectors (Mustapha and Dimitrakopoulos 2011b), that is, $T=\{{h}_{1},\dots ,{h}_{n}\}$. The template connects a specific location $x$ to each of the positions in the neighbourhood ${N}_{x}=x+T=\{{x}_{1},\dots ,{x}_{n}\}$. Without loss of generality, hereafter the real-valued spatial random field associated to the location $x$ and its neighbourhood ${N}_{x}$ is denoted by $Z(x,{N}_{x}),$ and its realization is denoted by $Z({x}_{i},{N}_{{x}_{i}})$. Furthermore, the data event vector ${d}_{{N}_{x}}=Z({N}_{x})=[Z({x}_{1}),\dots ,Z({x}_{n}){]}^{T}$ represents the realization of the field at the position of the neighbourhood nodes.

### Invariant High-Order Statistics

Vieta's formula (Funkhouser 1930) presents a relation between the coefficients and the roots of a polynomial. For a polynomial of degree $n,$

1 $$ p\left( X \right) = X^{n} + c_{1} u_{1} X^{n - 1} + \cdots + c_{n} u_{n} , $$

with the roots $[{x}_{1},\dots ,{x}_{n}{]}^{\mathrm{T}},$ the coefficients are $U=[{u}_{1},\dots ,{u}_{n}{]}^{\mathrm{T}},$ where

2 $$ u_{m} = \frac{1}{{c_{m} }}\mathop \sum \limits_{{\left( {1 \le i_{1} \le n - m + 1} \right)}} \mathop \sum \limits_{{\left( {i_{i} < i_{2} \le n - m + 2} \right)}} \ldots \mathop \sum \limits_{{\left( {i_{m - 1} < i_{m} \le n} \right)}} x_{{i_{1} }} \ldots x_{{i_{m} }} , $$

with $m\in \{1,\dots ,n\}$ and the normalization factor ${c}_{m}=(\genfrac{}{}{0pt}{}{n}{m}){\sigma }^{m}$, where $\sigma$ is the standard deviation of the $x$ in the TI. Furthermore, a recurrence relation could present the coefficients of a polynomial $q(X)=(X-{x}_{0})p(X)={X}^{n+1}+{c}_{1}{\overline{u}}_{1}{X}^{n}+\dots +{c}_{n+1}{\overline{u}}_{n+1}$, which has one extra root ${x}_{0}$, that is, $\{{x}_{0},{x}_{1},\dots ,{x}_{n}\}$. Then, it follows that

$$ \overline{u}_{m} = \frac{{c_{m - 1} }}{{c_{m} }}x_{0} + u_{m} $$

3 $$ m \in \left\{ {1, \ldots ,n + 1} \right\},\;\;c_{0} = 0,\;\;u_{n + 1} = 0. $$

Vieta's formula transforms the input vector of the roots of the polynomial $[{x}_{1},\dots ,{x}_{n}{]}^{\mathrm{T}}$ into the vector of the coefficients $[{u}_{1},\dots ,{u}_{n}{]}^{\mathrm{T}}$, with the advantage of being invariant to the ordering of the domain, given that the polynomial is invariant under a re-ordering of its roots. Herein, Eq. (2) is used to transform either the patterns or the data events of the simulation and TI, such that

$$ Z\left( {x_{s} ,N_{{x_{s} }} } \right) \to U_{{Z\left( {x_{s} ,N_{{x_{s} }} } \right)}} ,\;\;\;({\text{pattern}}\;{\text{simulation}}\;{\text{grid}}) $$

$$ Z\left( {x_{t} ,N_{{x_{t} }} } \right) \to U_{{Z\left( {x_{t} ,N_{{x_{t} }} } \right)}} ,\;\;({\text{pattern}}\;{\text{TI}}) $$

$$ d_{{N_{{x_{s} }} }} \to U_{{d_{{N_{{x_{s} }} }} }} ,\;\;({\text{data}}\;{\text{event}}\;{\text{simulation}}\;{\text{grid}}) $$

4 $$ d_{{N_{{x_{t} }} }} \to U_{{d_{{N_{{x_{t} }} }} }} ,\;\;({\text{data}}\;{\text{event}}\;{\text{TI}}). $$

### High-Order Statistics Distance Vector

Given two sets of data events $d_{{N_{{x_{s} }} }}$ and $d_{{N_{{x_{s} }} }}$ of order *n*, in order to develop an L2-norm distance measure, one must compare all possible ordering of these two data events, which results in *n *× (*n*!) number of operations. Hence, for a pattern of order *n *= 5, the number of operations becomes 600 per pattern, which is computationally expensive and can only operate on small TIs. In a new approach (Abolhassani et al. 2017), the L2-norm distance is calculated for the high-order statistics vectors of the data events

$$ D\left( {d_{{N_{{x_{s} }} }} ,d_{{N_{{x_{t} }} }} } \right) = U_{{d_{{N_{{x_{s} }} }} }} - U_{{d_{{N_{{x_{t} }} }} }} $$

5 $$ D\left( {Z\left( {x_{s} ,N_{{x_{s} }} } \right),Z\left( {x_{t} ,N_{{x_{t} }} } \right)} \right) = U_{{Z\left( {x_{s} ,N_{{x_{s} }} } \right)}} - U_{{Z\left( {x_{t} ,N_{{x_{t} }} } \right)}} , $$

where the high-order statistics of the data event $U$ s are calculated from Eq. (2). It is worth mentioning that the number of operations for this new distance measure is dramatically reduced to $n\times {2}^{n-1}$ per simulation node versus $n\times \left(n\right)!$ in L2-norm. For $n=5$, $\#\mathrm{op}(L2-\mathrm{norm})=600$ versus $\#\mathrm{op}(\mathrm{high}-\mathrm{ord})=80$.

###  Modelling the Spatial Non-Stationary Joint Cumulative Distribution Function (CDF) as a Finite Sum of Disjoint CDFs

Given a probability space, the real-valued spatial random field $Z(x,{N}_{x})$ is fully characterized by its non-stationary joint cumulative probability distribution function $F(x,Z(x,{N}_{x}))$. In this work, this function is modelled by the statistical ensemble (Landau and Lifshitz 1980) given by

6 $$ F\left( {x_{0} ,Z\left( {x,N_{x} } \right)} \right) = \mathop \sum \limits_{i = 1}^{m} \phi_{i} \left( {x_{0} } \right)F_{i} \left( {Z\left( {x,N_{x} } \right)} \right), $$

which is a sum over $m$ mutually exclusive stationary CDFs, ${F}_{i}$s, with a set of non-stationary mixing coefficients, known as a state set with binary variables ${\phi }_{i}({x}_{0})\in \{\mathrm{0,1}\}$. At any position ${x}_{0}$, the value of only one of the coefficients is 1, and the rest are zero, that is, ${\phi }_{k}=1$ and ${\phi }_{i}=0$ for $i\ne k$.

### Mutually Exclusive CDFs

On the grid of an input image with a fixed template *T*, Eq. (6) is considered to have a mutually exclusive set of stationary CDFs only if it satisfies the condition

$$ \exists \varepsilon \in R|\forall x_{1} ,x_{2} \in {\mathcal{D}} $$

$$ {\text{if}}\;\;\left| {D\left( {d_{{N_{{x_{1} }} }} ,d_{{N_{{x_{2} }} }} } \right)} \right| < \varepsilon $$

$$ \Rightarrow F\left( {x_{1} ,Z\left( {x,N_{x} } \right)} \right) = F\left( {x_{2} ,Z\left( {x,N_{x} } \right)} \right) = F_{k} \left( {Z\left( {x,N_{x} } \right)} \right) $$

$$ \Rightarrow {\text{The}}\;{\text{high - order}}\;{\text{statistics}}\;{\text{of}}\;{\text{two}}\;{\text{patterns}}\;{\text{match}} $$

7 $$ \left( {U_{{Z\left( {x_{1} ,N_{{x_{1} }} } \right)}} \approx U_{{Z\left( {x_{2} ,N_{{x_{2} }} } \right)}} } \right), $$

where *D*(.) is the high-order statistics distance vector calculated from Eq. (5) to compare two sets of high-order statistics vectors of two data events ${d}_{{N}_{{x}_{1}}}$ and ${d}_{{N}_{{x}_{2}}}$. In addition, ${U}_{Z({x}_{1},{N}_{{x}_{1}})}$ and ${U}_{Z({x}_{2},{N}_{{x}_{2}})}$ are the high-order statistics of two patterns, introduced in Sect. 2.3, with elements calculated from relation (2). According to Eq. (7), if the statistics of the data event of two patterns are close, then their CDFs are equal to an identical stationary CDF, defined as ${F}_{k}(Z(x,{N}_{x})), \mathrm{and}$, and further, that their high-order statistics are the same. This also implies that ${\phi }_{k}({x}_{1})={\phi }_{k}({x}_{2})=1$.

A set of mutually exclusive CDFs are used as the basis functions to span a more complex non-stationary CDF using Eq. (6). At each position on the grid, only one basis function is responsible for the simulation of the node; that is, each basis function ${F}_{k}(Z(x,{N}_{x}))$ simulates a set of nodes ${\mathcal{D}}_{k}$ on the grid. The ${\mathcal{D}}_{k}$ s are mutually disjoint, and their union is the entire grid $\mathcal{D}={\cup }_{k=1}^{m}{\mathcal{D}}_{k}$. As a result, for each simulation node, the entire TI is searched for nodes with similar statistics. Considering Eq. (7), the resulting set of nodes are generated from one of the basis CDFs and used for estimating the high-order statistics of that basis CDF, and the simulation node value is then sampled from the CDF.

### High-Order Transformation-Invariant Sequential Simulation

**Fig. 1:**
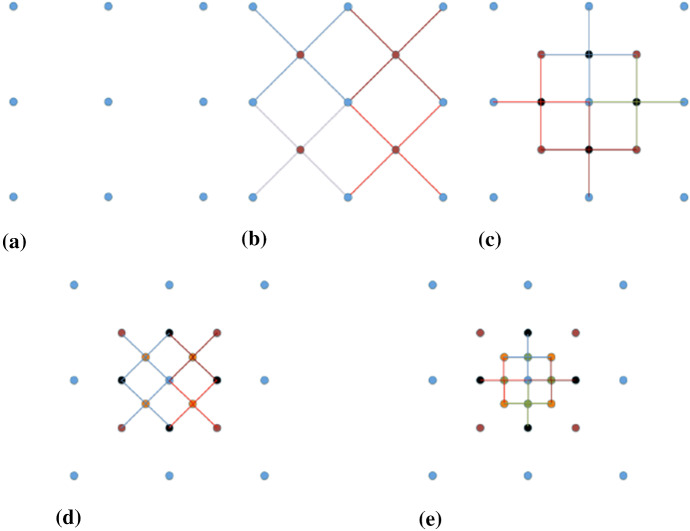
The hierarchy of the sequential multi-grid simulation. The order of the simulation starting from the blue nodes (sample data) and the red nodes (simulated first), followed by black nodes, orange nodes and eventually the green nodes. The simulation of each stage is conditioned on the previous simulated nodes

The goal is to simulate the non-stationary, real-valued random field $\{Z({x}_{s},{N}_{{x}_{s}})|{x}_{s}\in {\Omega }_{s}\}$ at the position of all simulation nodes ${\Omega }_{s}$ with a fixed template T. A sequential multi-grid process is used when provided with a sparse set of data $\{Z({x}_{h},{N}_{{x}_{h}})|{x}_{h}\in {\Omega }_{h}\}$ on a regular grid ${\Omega }_{h}$ and a fine-resolution TI $\{Z({x}_{t},{N}_{{x}_{t}})|{x}_{t}\in {\Omega }_{t}\}$. In the hierarchy of the sequential multi-grid simulation, the coarse templates are firstly adopted to complete the first-stage simulation. As more nodes are simulated, finer-scale templates are used to search for the conditioning data. The order of the sequential simulation is shown in Fig. 1.

At each sequence, the path is chosen randomly and saved into a vector containing the indices of the visiting nodes ${I}_{s}$. Each successive random variable $Z({x}_{s},{N}_{{x}_{s}})$ at node $\{{x}_{s}|s\in {I}_{s}\}$ is conditioned to a data event ${d}_{{N}_{{x}_{s}}}$ and the neighbourhood nodes that are selected from the set of previously simulated nodes and the sample data (Goovaerts 1997). A template $T=\{{h}_{1},\dots ,{h}_{n}\}$ is formed spatially by connecting each data event node to the simulation node. The first goal is to simulate the high-order statistics of the pattern, that is, ${U}_{Z({x}_{s},{N}_{{x}_{s}})}$, and then to sample the simulation node $Z\left({x}_{s},{N}_{{x}_{s}}\right),$ given the CPDF $P({U}_{Z({x}_{s},{N}_{{x}_{s}})}|{U}_{{d}_{{N}_{{x}_{s}}}},{U}_{Z({x}_{t},{N}_{{x}_{t}})})$. In this case, the ${U}_{(.)}$ function refers to the high-order statistics vector with the elements calculated from Eq. (2). This probability is complex and cannot be simulated directly unless it is represented by a model with a set of parameters $\theta \in\Theta$ that are independent from the data event and TI. The parameter $\theta$ is optimized to express the data event and TI. Thus, this probability can be decomposed using the total probability rule into

8 $$\begin{aligned}& P\left( {U_{{Z\left( {x_{s} ,N_{{x_{s} }} } \right)}} |U_{{d_{{N_{{x_{s} }} }} }} ,U_{{Z\left( {x_{t} ,N_{{x_{t} }} } \right)}} } \right)\\ &\quad = \int\limits_{\theta \in \Theta } {P\left( {U_{{Z\left( {x_{s} ,N_{{x_{s} }} } \right)}} |\theta ,U_{{d_{{N_{{x_{s} }} }} }} ,U_{{Z\left( {x_{t} ,N_{{x_{t} }} } \right)}} } \right)P\left( {\theta |U_{{d_{{N_{{x_{s} }} }} }} ,U_{{Z\left( {x_{t} ,N_{{x_{t} }} } \right)}} } \right){\text{d}}\theta } .\end{aligned} $$

**Fig. 2:**
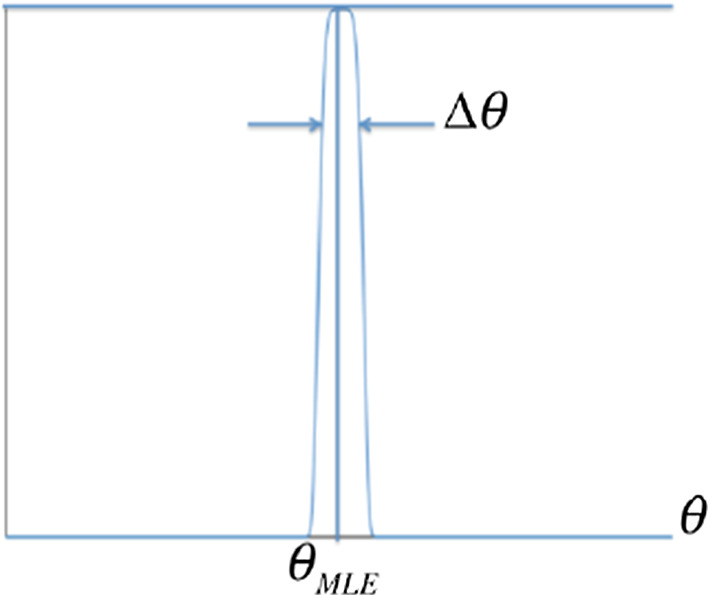
The representation of $P\left( {\theta |U_{{d_{{N_{{x_{s} }} }} }} ,U_{{Z\left( {x_{t} ,N_{{x_{t} }} } \right)}} } \right)$ as a function of $\theta$

An estimation of the argument of the integral further simplifies Eq. (8). Figure 2 represents the term $P(\theta |{U}_{{d}_{{N}_{{x}_{s}}}},{U}_{Z({x}_{t},{N}_{{x}_{t}})})$ as a function of $\theta$. The contribution of this function is negligible except for a narrow band near an optimal value for the parameter’s maximum a posteriori estimation (MAP) ${\theta }_{\mathrm{MAP}}$. Consequently, Eq. (8) can be estimated as

9 $$ \begin{aligned}& P\left( {U_{{Z\left( {x_{s} ,N_{{x_{s} }} } \right)}} |U_{{d_{{N_{{x_{s} }} }} }} ,U_{{Z\left( {x_{t} ,N_{{x_{t} }} } \right)}} } \right)\\&\quad \approx  P\left( {U_{{Z\left( {x_{s} ,N_{{x_{s} }} } \right)}} |\theta_{{{\text{MAP}}}} ,U_{{d_{{N_{{x_{s} }} }} }} ,U_{{Z\left( {x_{t} ,N_{{x_{t} }} } \right)}} } \right)\overbrace {{P\left( {\theta_{{{\text{MAP}}}} |U_{{d_{{N_{{x_{s} }} }} }} ,U_{{Z\left( {x_{t} ,N_{{x_{t} }} } \right)}} } \right)\Delta \theta }}^{{{\text{constant}}}} \\&\quad  =  \frac{1}{{C_{{{\text{MAP}}}} }}P\left( {U_{{Z\left( {x_{s} ,N_{{x_{s} }} } \right)}} |\theta_{{{\text{MAP}}}} ,U_{{d_{{N_{{x_{s} }} }} }} ,U_{{Z\left( {x_{t} ,N_{{x_{t} }} } \right)}} } \right). \end{aligned} $$

In Eq. (9), ${C}_{\mathrm{MAP}}$ is the normalization factor to ensure the validity of the probability. Equation (9) implies that the probability distribution function of the node ${x}_{s}$ can be calculated if ${\theta }_{\mathrm{MAP}}$ is known. To estimate ${\theta }_{\mathrm{MAP}}$ based on (Fig. 2), $\theta ={\theta }_{\mathrm{MAP}}$ when $P(\theta |{U}_{{d}_{{N}_{{x}_{s}}}},{U}_{Z({x}_{t},{N}_{{x}_{t}})})$ is maximized for $\theta \in\Theta$; that is,

10 $$ \theta_{{{\text{MAP}}}} = \mathop {\arg \max }\limits_{\theta \in \Theta } P\left( {\theta |U_{{d_{{N_{{x_{s} }} }} }} ,U_{{Z\left( {x_{t} ,N_{{x_{t} }} } \right)}} } \right). $$

Using Bayes’ rule, the right-hand side of the equation is extended into

11 $$ \theta_{{{\text{MAP}}}} = \mathop {\arg \max }\limits_{\theta \in \Theta } \frac{{P\left( {U_{{Z\left( {x_{t} ,N_{{x_{t} }} } \right)}} |\theta ,U_{{d_{{N_{{x_{s} }} }} }} } \right)\overbrace {{P\left( {\theta |U_{{d_{{N_{{x_{s} }} }} }} } \right)}}^{{{\text{uniform}}\;{\text{prior}}}}}}{{\underbrace {{P\left( {U_{{Z\left( {x_{t} ,N_{{x_{t} }} } \right)}} |U_{{d_{{N_{{x_{s} }} }} }} } \right)}}_{{{\text{independent}}\;{\text{from}}\;\theta }}}}. $$

In this case, one assumes a uniform prior in the parameter space $\Theta$, with the marginal probability in TI $P({U}_{Z({x}_{t},{N}_{{x}_{t}})})$ remaining independent from $\theta$. Hence, the ${\theta }_{\mathrm{MAP}}$ is equivalent to the maximum likelihood estimation ${\theta }_{\mathrm{MLE}},$ and Eq. (11) simplifies into

12 $$ \theta_{{{\text{MLE}}}} = \mathop {\arg \max }\limits_{\theta \in \Theta } P\left( {U_{{Z\left( {x_{t} ,N_{{x_{t} }} } \right)}} |\theta ,U_{{d_{{N_{{x_{s} }} }} }} } \right). $$

Each node in TI is only conditioned on its neighbours ${N}_{{x}_{t}}$ and the parameter set $\theta$. Hence, the joint distribution in Eq. (12) can be further decomposed into

13 $$ \theta_{{{\text{MLE}}}} = \mathop {\arg \max }\limits_{\theta \in \Theta } \mathop \prod \limits_{{x_{t} \in \Omega_{t} }} P\left( {U_{{Z\left( {x_{t} ,N_{{x_{t} }} } \right)}} |\theta ,U_{{d_{{N_{{x_{s} }} }} }} ,U_{{d_{{N_{{x_{t} }} }} }} } \right). $$

A probability is maximized if the logarithm of that probability is maximized.

14 $$ \theta_{{{\text{MLE}}}} = \mathop {\arg \max }\limits_{\theta \in \Theta } \mathop \sum \limits_{{x_{t} \in \Omega_{t} }} \log P\left( {U_{{Z\left( {x_{t} ,N_{{x_{t} }} } \right)}} |\theta ,U_{{d_{{N_{{x_{s} }} }} }} ,U_{{d_{{N_{{x_{t} }} }} }} } \right). $$

Furthermore, at the maximum, the derivative with respect to $\theta$ should be zero; that is,

15 $$ \frac{\partial }{\partial \theta }\left( {\mathop \sum \limits_{{x_{t} \in \Omega_{t} }} \log P\left( {U_{{Z\left( {x_{t} ,N_{{x_{t} }} } \right)}} |\theta ,U_{{d_{{N_{{x_{s} }} }} }} ,U_{{d_{{N_{{x_{t} }} }} }} } \right) } \right) = 0. $$

Equation (15) is solved for an optimal solution ${\theta }_{\mathrm{MLE}}$ by modelling the $P({U}_{Z({x}_{t},{N}_{{x}_{t}})}|\theta ,{U}_{{d}_{{N}_{{x}_{s}}}},{U}_{{d}_{{N}_{{x}_{t}}}})$ with an exponential family in Sect. 2.8.

### High-Order Simulation Model

The exponential family is used here to model the likelihood function in Eq. (15) with the parameter set $\theta =[{\theta }_{1},\dots ,{\theta }_{n+1}{]}^{T}$ as

16 $$ \begin{aligned} P\left( {U_{{Z\left( {x_{t} ,N_{{x_{t} }} } \right)}} |\theta ,U_{{d_{{N_{{x_{s} }} }} }} ,U_{{d_{{N_{{x_{t} }} }} }} } \right) = & \int\limits_{0}^{1} {\overbrace {{\exp \left( { - W(U_{{Z\left( {x_{t} ,N_{{x_{t} }} } \right)}} - \theta )^{T} \left( {U_{{Z\left( {x_{t} ,N_{{x_{t} }} } \right)}} - \theta } \right)} \right)}}^{{P\left( {U_{{Z\left( {x_{t} ,N_{{x_{t} }} } \right)}} |W,\theta ,U_{{d_{{N_{{x_{s} }} }} }} ,U_{{d_{{N_{{x_{t} }} }} }} } \right)}}} P\left( W \right){\text{d}}W \\ = & \frac{1}{{\sqrt {2\pi \sigma_{0}^{2} } }}\exp \left( { - W_{ML} (U_{{Z\left( {x_{t} ,N_{{x_{t} }} } \right)}} - \theta )^{T} \left( {U_{{Z\left( {x_{t} ,N_{{x_{t} }} } \right)}} - \theta } \right)} \right). \\ \end{aligned} $$

In Eq. (16), $W$ is introduced as a weight function based on the similarity measure of the data event ${d}_{{N}_{{x}_{s}}}$ and ${d}_{{N}_{{x}_{t}}}$. It ensures that the TI patterns with more similar data events contribute more to the likelihood function. $W$ has a probability distribution over $[0, 1]$; ${W}_{\mathrm{ML}}$ is the maximum likely similarity that is estimated by

$$ W_{{{\text{ML}}}} \cong \omega \left( {d_{{N_{{x_{s} }} }} ,d_{{N_{{x_{t} }} }} } \right) = \exp \left( { - \frac{{D\left( {d_{{N_{{x_{s} }} }} ,d_{{N_{{x_{t} }} }} } \right)^{{\text{T}}} D\left( {d_{{N_{{x_{s} }} }} ,d_{{N_{{x_{t} }} }} } \right)}}{{\left| {\sqrt {\Sigma \left( {d_{{N_{{x_{t} }} }} } \right)} } \right|}}} \right), $$

17 $$ \Sigma \left( {d_{{N_{{x_{t} }} }} } \right) = \frac{1}{{N^{2} }}\mathop \sum \limits_{{x_{t} \in \Omega_{t} }} \mathop \sum \limits_{{y_{t} \in \Omega_{t} }} \left( {d_{{N_{{x_{t} }} }} - d_{{N_{{y_{t} }} }} } \right)^{2} , $$

where $D(.,.)$ is given by Eq. (5) as the high-order statistics distance vector, a distance measure between two data events, and $\Sigma \left({d}_{{N}_{{x}_{t}}}\right)$ is the mean square distance vector of the data event over the TI. Note that the weight $W$ is defined to be a positive function that decreases as the distance between the two data events increases. ${{\sigma }_{0}}^{2}$ in Eq. (16) is to ensure that a proper probability is established for Eq. (16).

18 $$ \int\limits_{ - \infty }^{\infty } {\int\limits_{0}^{1} {P\left( {U_{{Z\left( {x_{t} ,N_{{x_{t} }} } \right)}} |W,\theta ,U_{{d_{{N_{{x_{s} }} }} }} ,U_{{d_{{N_{{x_{t} }} }} }} } \right)P\left( W \right){\text{d}}W{\text{d}}u = 1.} } $$

Substituting Eq. (16) into Eq. (15), the solution of the ${\theta }_{\mathrm{MLE}}$ is

19 $$ \theta_{{{\text{MLE}}}} = \frac{{\mathop \sum \nolimits_{{x \in \Omega_{t} }} \omega \left( {d_{{N_{{x_{s} }} }} ,d_{{N_{{x_{t} }} }} } \right)U_{{Z\left( {x_{t} ,N_{{x_{t} }} } \right)}} }}{{\mathop \sum \nolimits_{{x \in \Omega_{t} }} \omega \left( {d_{{N_{{x_{s} }} }} ,d_{{N_{{x_{t} }} }} } \right)}}, $$

the non-stationary expected value of the vector of the high-order statistics of the simulation pattern. Having calculated the ${\theta }_{\mathrm{MLE}}=[{\theta }_{1},\dots ,{\theta }_{n+1}]$ in Eq. (19) and considering Eq. (9), one gets

20 $$\begin{aligned} & P\left( {U_{{Z\left( {x_{s} ,N_{{x_{s} }} } \right)}} |U_{{d_{{N_{{x_{s} }} }} }} ,U_{{Z\left( {\Omega_{t} ,N_{{\Omega_{t} }} } \right)}} } \right)\\ &\quad \approx \frac{1}{{\sqrt {2\pi } }}\exp \left( { - \frac{1}{2}\left( {U_{{Z\left( {x_{s} ,N_{{x_{s} }} } \right)}} - \theta_{MLE} } \right)^{T} \left( {U_{{Z\left( {x_{s} ,N_{{x_{s} }} } \right)}} - \theta_{MLE} } \right)} \right).\end{aligned} $$

Applying the recurrence relation (3), Eq. (20) is rewritten in terms of the simulation data event ${U}_{{d}_{{N}_{{x}_{s}}}}$ and the simulation node $Z({x}_{s})$ (the extra root in Vieta's formula (3)); that is,

21 $$ \begin{aligned} P\left( {Z\left( {x_{s} } \right)|d_{{N_{{x_{s} }} }} ,d_{{N_{{x_{t} }} }} } \right) \approx & \frac{1}{{\sqrt {2\pi \delta_{n + 1}^{2} } }}\exp \left( { - \frac{{\left( {Z\left( {x_{s} } \right) - \mu_{n + 1} \left( {\theta_{{{\text{MLE}}}} } \right)} \right)^{2} }}{{2\delta_{n + 1}^{2} }}} \right) \\ \frac{1}{{\delta_{n + 1}^{2} }} = & \sum\limits_{m = 1}^{n + 1} {\left( {\frac{{u_{m - 1} c_{m - 1} }}{{c_{m} }}} \right)^{2} } \\ \mu_{n + 1} \left( {\theta_{{{\text{MLE}}}} } \right) = & \delta_{n + 1}^{2} \sum\limits_{m = 1}^{n + 1} {\frac{{\left( {u_{m} - \theta_{m} } \right)u_{m - 1} c_{m - 1} }}{{c_{m} }}} , \\ \end{aligned} $$

where ${u}_{m}$ can be calculated with Eq. (2) and ${c}_{m}=(\genfrac{}{}{0pt}{}{n}{m}){\sigma }^{m}$, in which $\sigma$ is the standard deviation of the $x$ in the TI. Eventually, the simulation node $Z({x}_{s})$ can be sampled from the distribution in Eq. (21).

### Algorithm

1. Define a random path ${\overline{\Omega }}_{s}$ to visit all positions on the simulation grid ${\Omega }_{s}$.
2. Select the next unsampled position on the simulation grid $x_{s} \in {\overline{\Omega }}_{s}$.
3. Search the conditioning data ${\Omega }_{h}$ for $n$ immediate neighbourhood positions $N_{{x_{s} }} = \left\{ {x_{1} , \ldots ,x_{n} } \right\} \subset {\Omega }_{h}$ of the simulation node $x_{s}$.
4. Generate the data event vector $d_{{N_{{x_{s} }} }} = Z\left( {N_{{x_{s} }} } \right) = [Z\left( {x_{1} } \right), \ldots ,Z\left( {x_{n} } \right)]^{T}$ with the node values at the neighbourhood positions $N_{{x_{s} }}$.
5. Compute the high-order statistics vector of the data event $U_{{d_{{N_{{x_{s} }} }} }} = [u_{1} , \ldots ,u_{n} ]^{T}$ with elements $u_{m}$ calculated from Eq. (2).
6. Generate template $$T = \left\{ {h_{1} , \ldots ,h_{n} } \right\} = \left\{ {x_{1} - x_{s} , \ldots ,x_{n} - x_{s} } \right\}$$.
7. Search all positions in the TI and select the positions of the patterns $\left( {{\Omega }_{t} ,N_{{{\Omega }_{t} }} } \right)$ matching the template T.
8. Select the next unvisited TI pattern $\left( {x_{t} ,N_{{x_{t} }} } \right) \in \left( {{\Omega }_{t} ,N_{{{\Omega }_{t} }} } \right)$.
9. Generate the TI data event $d_{{N_{{x_{t} }} }} = Z\left( {N_{{x_{t} }} } \right) = [Z\left( {y_{1} } \right), \ldots ,Z\left( {y_{n} } \right)]^{T}$ according to the node values in the TI.
10. Calculate the high-order statistics vector $U_{{d_{{N_{{x_{t} }} }} }} = [u_{1} , \ldots ,u_{n} ]^{T}$ of the data event $d_{{N_{{x_{t} }} }}$ with elements $u_{m}$ calculated from Eq. (2).
11. Calculate the high-order distance $D\left( {d_{{N_{{x_{s} }} }} ,d_{{N_{{x_{t} }} }} } \right)$ in Eq. (5).
12. Calculate the similarity measure ${\Sigma }\left( {d_{{N_{{x_{t} }} }} } \right)$ from Eq. (17).
13. Continue to Step (8) until all of the TI patterns are visited.
14. Calculate $\theta_{MLE} = [\theta_{1} , \ldots ,\theta_{n + 1} ]^{T}$ using Eq. (19).
15. Draw a sample $\overline{Z}\left( {x_{s} } \right)$ randomly from the distribution function in Eq. (21).
16. Continue to Step (2) until all of the grid is simulated.

## Examples and Comparisons

Applied aspects of the simulation method presented in the previous section are examined in this section using two-dimensional horizontal layers from the Stanford V fluvial reservoir (Mao and Journel 1999). Selected layers represent exhaustive images that are then sampled to generate data sets subsequently used to generate simulations with the proposed new non-stationary high-order spatial transformation-invariant simulation method, HOSTSIM. The statistics of the simulated realizations, namely, histogram, variogram map and third-order L-shaped cumulant map, are then compared to the ones calculated from the initial data. In addition, the simulations generated by the HOSTSIM method are compared to those generated by the well-known FILTERSIM multiple-point simulation method (Zhang et al. 2006) through its implementation available in the public domain (Remy et al. 2009).

**Fig. 3:**
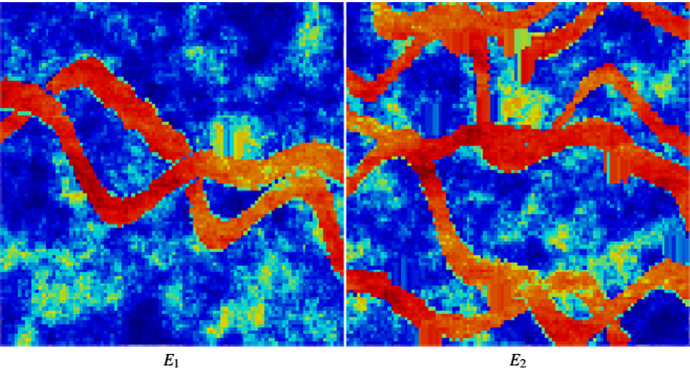
From left to right, the exhaustive images $E_{1}$ and $E_{2}$ (ground truth)

**Fig. 4:**
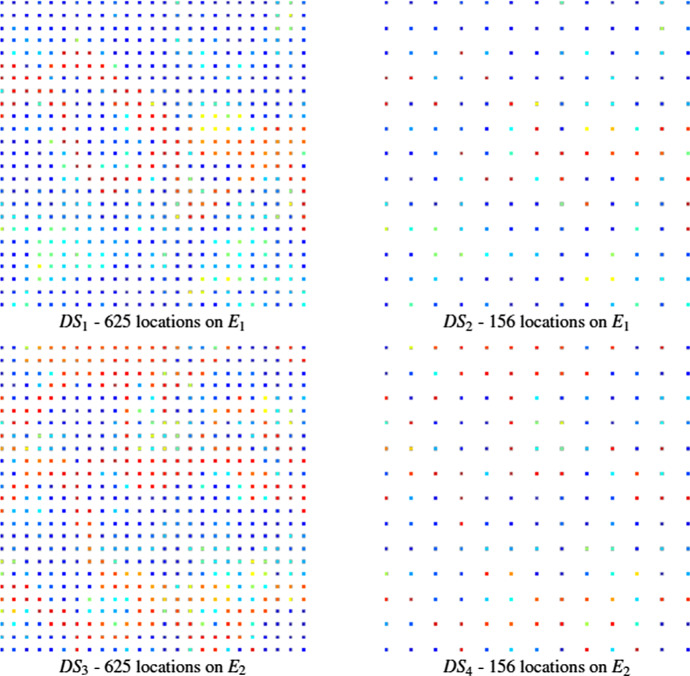
Data samples used for the simulations

**Fig. 5:**
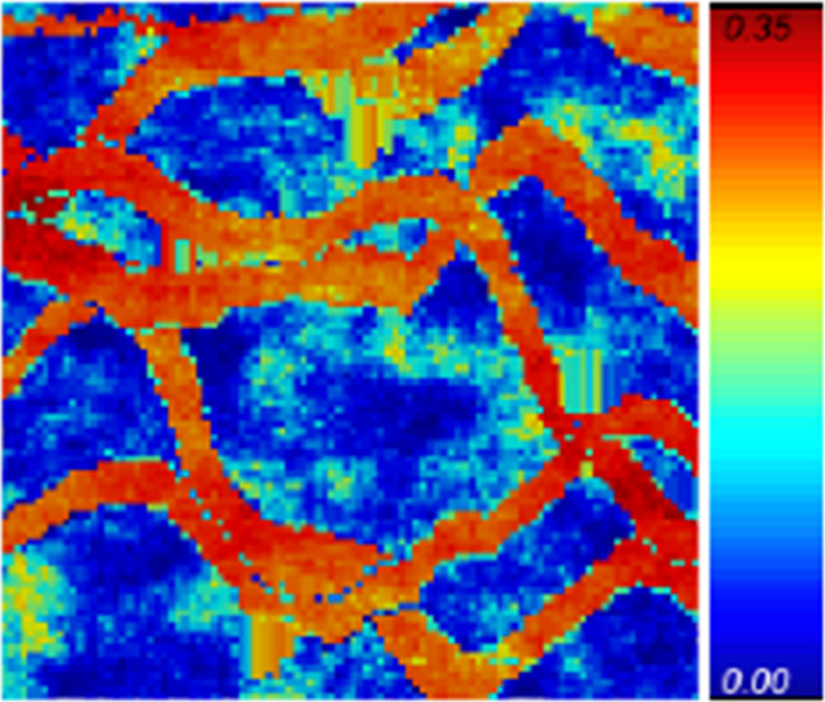
TI used in all four simulation cases

Figure 3 shows two exhaustive images used for the simulations $$ E_{1}$$ and $$E_{2}$$; each one is a horizontal section of the complete data with size of $$100 \times 100m^{2}$$. Two sets of data are then selected from each section $$E_{1}$$ and $$E_{2}$$, one sampled at 625 and the other at 156 locations, respectively, resulting in four different data sets, namely $$DS_{1}$$ (625 points from $$E_{1}$$), $$DS_{2}$$ (156 points from $$E_{1}$$), $$DS_{3}$$ (625 points from $$E_{2}$$) and $$DS_{4}$$ (156 points from $$E_{2}$$), as shown in Fig. 4. The TI used for all simulations is a different section selected from the Stanford V fluvial reservoir and is shown in Fig. 5.

**Fig. 6:**
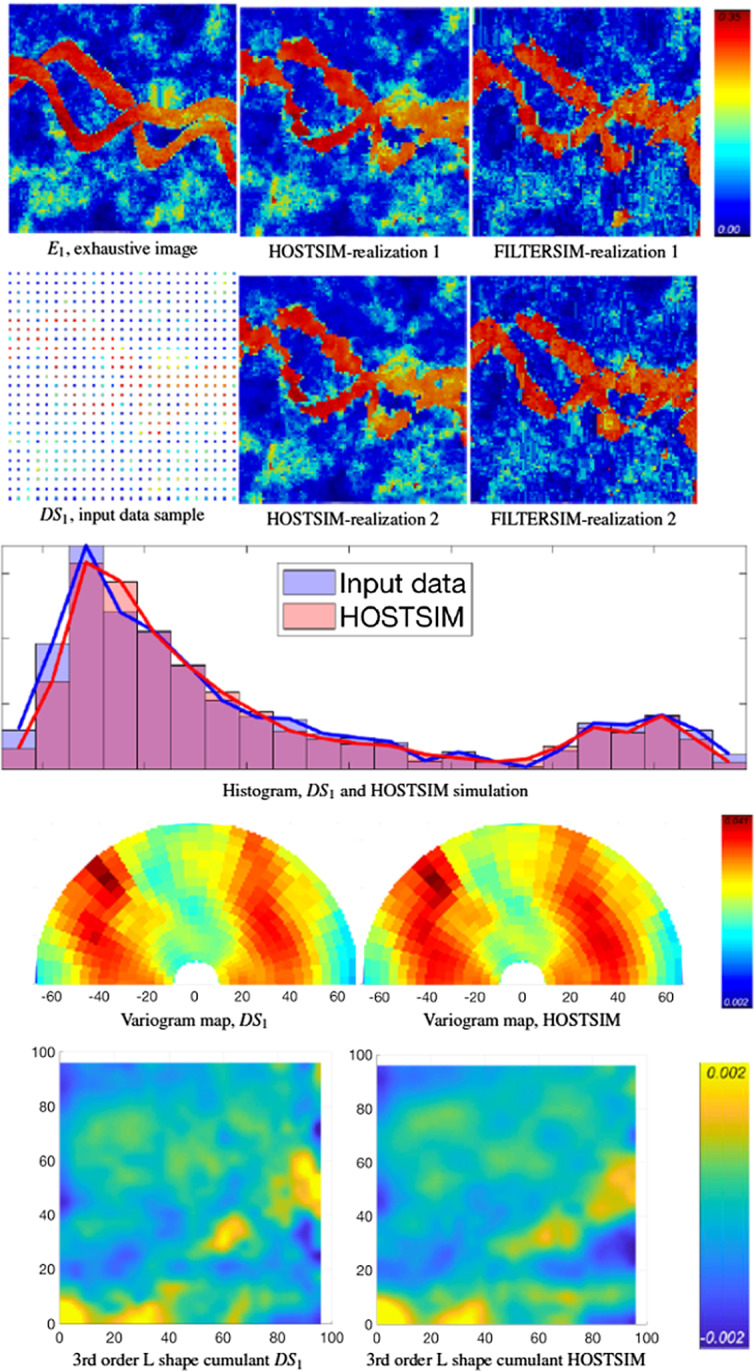
Example 1: The $E_{1}$ exhaustive image is provided in the top row (left), and the input image $DS_{1}$ is in the second row. Two HOSTSIM and FILTERSIM simulations are in the second and third columns (top section), followed by the histogram, variogram map and third-order spatial cumulant map below

**Fig. 7:**
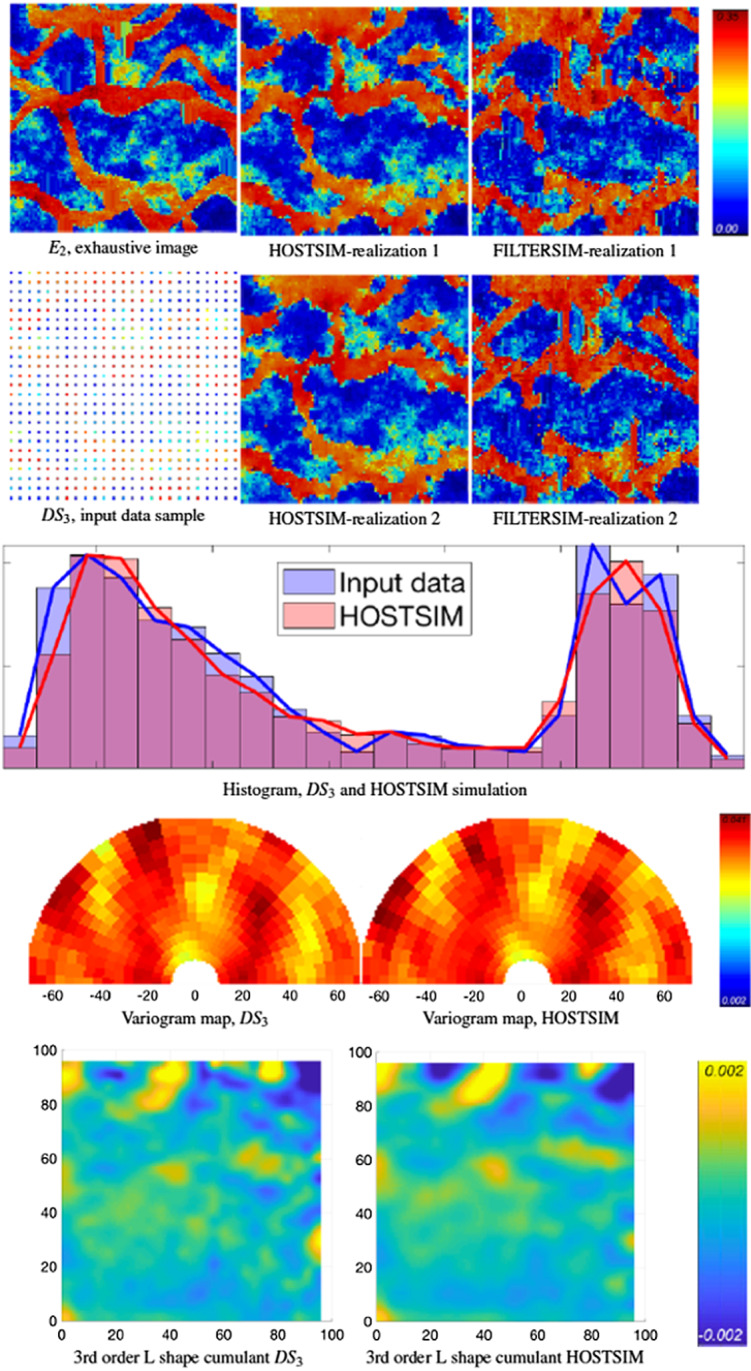
Example 1: The $E_{2}$ exhaustive image is shown in the top row (left), and the input image $DS_{3}$ is in the second row. The two HOSTSIM and FILTERSIM simulations are in the second and third columns (above), followed by the histogram, variogram map and third-order spatial cumulant map below

**Fig. 8:**
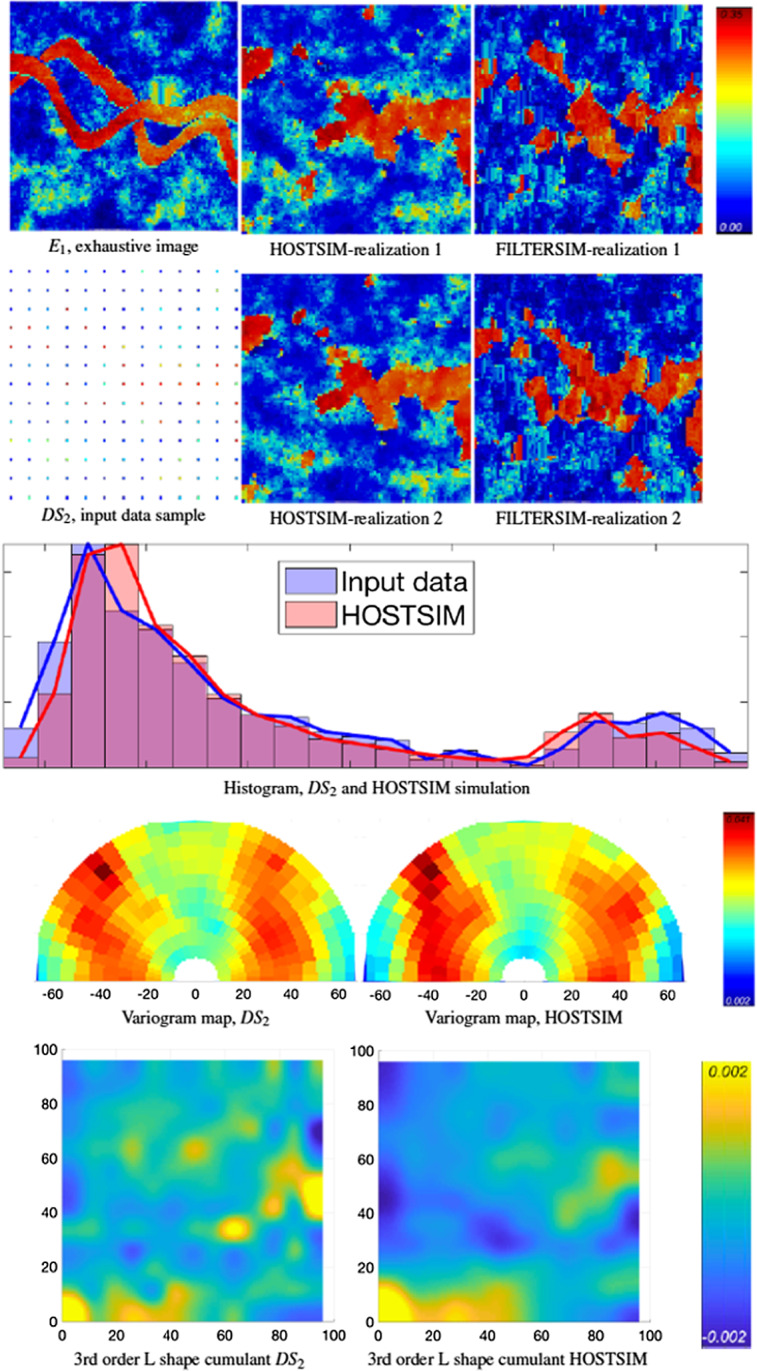
Example 2: The $E_{1}$ exhaustive image is in top row (left), and the input image $DS_{2}$ is in the second row. The two HOSTSIM and FILTERSIM simulations are in the second and third columns (above), followed by the histogram, variogram map and third-order spatial cumulant map (below)

**Fig. 9:**
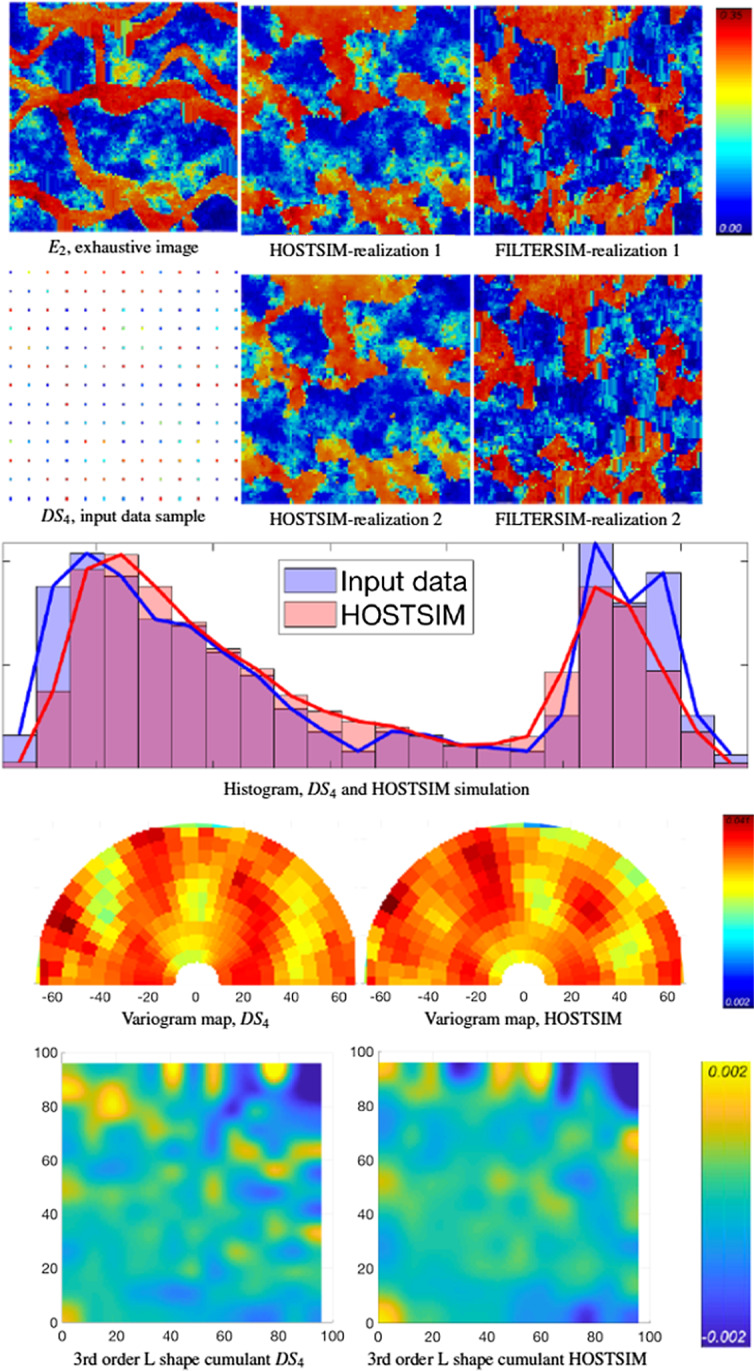
Example 2: The $E_{2}$ exhaustive image is shown in the top row (left), and the input image $DS_{4}$ is provided in the second row. The two HOSTSIM and FILTERSIM simulations are in the second and third columns (above), followed by the histogram, variogram map and third-order spatial cumulant map (below)

In each example, one of the data sets in Fig. 4 and the TI in Fig. 5 are used as input for simulations, and two realizations are generated by each method noted above. The exhaustive images, the input data and the realizations generated by HOSTSIM and FILTERSIM are illustrated in Figs. 6, 7, 8 and 9 for data samples $DS_{1}$ through $DS_{4}$, along with their respective validation graphs. The validation graphs are generated for both the input data and the HOSTSIM simulation and include the histogram, variogram map and third-order L-shaped spatial cumulant map. Note that the second-order variogram map of an image represents the variogram for each lag direction, that is, $r \in \left[ {0,65} \right]$ and $\theta \in \left\{ {0,\frac{\pi }{30}, \ldots ,\pi } \right\}$, in a polar coordinate system. The third-order L-shaped spatial cumulant of an image is generated by using an L-shaped template, which represents two orthogonal lag vectors from a common point. For all possible pairs of horizontal and vertical lags, the template is shifted over the image to extract the third-order cumulant by averaging the values calculated for each pattern within the image (Mustapha and Dimitrakopoulos 2010b, 2011a).

### Example 1

Figures 6 and 7 show the results generated from $DS_{1}$ and $DS_{3}$ with 625 data samples. There are low-contrast regions in the HOSTSIM realizations (light blue/green area seen toward the bottom of $E_{1}$ in Fig. 6, and in the bottom-right and top-center of the $E_{2}$ realization in Fig. 7) in both realizations. This behaviour is expected, as a denser set of data is available in these regions, and HOSTSIM can match similar patterns within the TI and better simulate the corresponding nodes. This is also the case in the high-value areas on the simulated realization (red-coloured regions, or channels). In general, both HOSTSIM and FILTERSIM perform well in reproducing the horizontal channels, which are along the preferential direction in both the exhaustive image and the denser data samples. However, HOSTSIM shows better performance than FILTERSIM in reproducing the connectivity along the vertical channels, as can be seen from the major vertical channels in the left part of the exhaustive image and the related realizations. In this situation, the concept of weighted high-order spatial statistics plays an important role; accordingly, HOSTSIM demonstrates an advantage because the samples along the vertical channels are given more weight according to their similarity to the sample data events even when they are less frequent. Although there are some cases where the connectivity of the channels is not well generated, for example in the top-right part of both realizations in Fig. 7, the narrow channel is not well represented in the data, and the HOSTSIM cannot accurately estimate the high-order statistic of the data event to match appropriate patterns from either the data or the TI. The histogram and variogram graphs of the HOSTSIM simulations are similar to those calculated for $DS_{1}$ and $DS_{3}$ in both Figs. 6 and 7. Similarity of the third-order spatial cumulants can also be observed from Figs. 6 and 7.

### Example 2

The HOSTSIM realizations are presented in Figs. 8 and 9. As the number of input data are reduced to 156 samples, the quality of the results is reduced (relative to Example 1). The low-contrast regions are, however, simulated in these examples (light blue/green areas). Some parts of the channels are not generated in both realizations (the center-left part of the channel in Fig. 8 and the center-left and bottom-left part of the channels in Fig. 9) due to a lack of high-value samples in those regions, which are necessary in order to represent narrow channels. On the other hand, some channels are merged in the HOSTSIM realizations (center-right part of both Figs. 8 and 9), which is due to a lack of low-value samples between two channels in the input data samples. The validation graphs of the HOSTSIM simulations are similar to the ones generated for the input data samples in Figs. 8 and 9, although they are somewhat degraded due to the sparsity of the input data. While the HOSTSIM realizations generated for this example are somewhat degraded compared with the previous example, there is still better connectivity in the data than in the results from FILTERSIM; for example, the channel in the center-right part of Fig. 8 is partially generated in HOSTSIM realizations but not well presented in the FILTERSIM realizations. Low-contrast regions are better represented in the HOSTSIM realizations as well (see the bottom-left part of Fig. 8). Regarding the reproduction of high-order spatial statistics, the third-order L-shaped spatial cumulants are well generated for the lags < 50 m. Considering that replicates with lags larger than 50 m are relatively few because the samples are taken from an area of 100 $\times$ 100 m^2^, given the sparsity of the samples, the inference of spatial cumulants within a short-scale range is more reliable in terms of comparisons.

To complete our analysis, two commonly used similarity measures in computer vision are applied here to compare the realizations generated by HOSTSIM and FILTERSIM for all cases. These measures are the peak signal-to-noise ratio (PSNR) and the structural similarity index (SSIM) (Salomon 2007). The PSNR is a point-to-point difference measure between two images in the logarithmic scale; in particular, the realization image is compared with the exhaustive image from point to point. Realizations that are more similar to the exhaustive image result in greater values, and where PSNR values approach infinity, the images are identical. Hence, this measure is useful for qualitatively comparing two realizations generated for an exhaustive image, as the PSNR values do not have an absolute interpretation. The SSIM, on the other hand, provides an absolute quantity comparing the similarity between a realization and an exhaustive image with a value between 0 and 1, where SSIM = 1 indicates identical images. Table 1 presents the PSNR and SSIM results calculated for HOSTSIM and FILTERSIM realizations generated for all four data sets ($$DS_{1}$$, $$DS_{2}$$, $$DS_{3}$$ and $$DS_{4} )$$. The reference exhaustive image for each data set is defined as the reference image in the calculation of PSNR and SSIM. HOSTSIM realizations are more similar to the exhaustive images in all cases. The SSIM values of the HOSTSIM realizations are $$\approx 10{\text{\% }}$$ more accurate than the FILTERSIM realizations.

**Table 1:** PSNR (left) and SSIM (right) values for all four cases

Case	HOSTSIM	FILTERSIM	CASE	HOSTSIM	FILTERSIM
*DS*_1_	24.94	23.10	*DS*_1_	0.61	0.50
*DS*_3_	23.10	21.29	*DS*_3_	0.56	0.42
*DS*_2_	21.23	20.98	*DS*_2_	0.47	0.43
*DS*_4_	20.17	18.76	*DS*_4_	0.40	0.3

## Conclusions

A new high-order, non-stationary stochastic simulation method was introduced in this paper. Given a spatially sparse set of data and a TI, this method simulates realizations of attributes of interest on a finer spatial grid. The method is designed to use only training patterns that respect the local high-order statistics of the simulation pattern by comparing the spatial high-order statistics of the data events of both the training and the target simulation patterns.

The high-order statistics of the data event of each simulation pattern is first calculated and compared with high-order statistics of the data event calculated for the training patterns with a similar spatial template. The contribution of each training pattern in simulating a pattern is determined by a weight calculated based on the similarity of the high-order statistics of the training and simulation patterns. The calculated weights are designed to be invariant against ordering the data event nodes. The high-order statistics of the simulation pattern is then estimated by calculating the non-stationary maximum likelihood estimate over the training patterns by giving more weight to the most similar patterns, after which a sample is drawn from the distribution function fitted to the estimated high-order statistics. The use of the high-order statistics in this simulation method ensures the ability to capture and reproduce complex patterns in the generated simulations. Meanwhile, using the non-stationarity similarity measure ensures that only training patterns that respect the local statistics of the simulation are used in the estimation of the high-order spatial statistics of the pattern. Notably, (i) non-stationary weight reduces the numerical instabilities that may be encountered in stationary high-order simulation methods, and (ii) the transformation-invariant quality of the measure ensures that different orientations of each TI are considered for the simulation of the pattern. The latter allows for data-driven simulations even without the use of a TI. Two set of examples have been used for testing the proposed method. The first contains 625 data points and the second contains a very sparse set of 156 data points. The simulation results are calculated on a 100 × 100 spatial grid. The training patterns are extracted from a 100 × 100 grid image. Despite the sparsity of the data in both examples, the method is able to simulate the channels in the initial exhaustive image. This is due to the utilization of a non-stationary rotation invariant weighting for the contribution of the patterns based on the similarity of the high-order statistics of their data events.

## Appendix: Similarity Measures

PSNR and SSIM (Salomon 2007) are two image similarity measures that are used in this paper to evaluate the simulation quality. PSNR (peak signal-to-noise ratio) is a measure calculated by comparing a reconstructed image to the original image. Assuming two $m \times n$ images denoted $I$ (reference) and $K$ (reconstructed), first the mean-square error is calculated according to the following equation

22 $$ {\text{MSE}} = \frac{1}{mn}\mathop \sum \limits_{i = 0}^{m - 1} \mathop \sum \limits_{j = 0}^{n - 1} \left[ {I\left( {i,j} \right) - K\left( {i,j} \right)} \right]^{2}. $$

The PSNR is then calculated as

23 $$ {\text{PSNR}} = 10\log \left( {\frac{{\max (I)^{2} }}{{{\text{MSE}}}}} \right). $$

This function returns a real value between zero and infinity. Greater values of PSNR imply better quality.

The structural similarity index (SSIM) is another quality measure used in this paper; it evaluates similarity according to a relative measure varying between 0 and 1 that compares an image to a reference image. Images that are more similar to the reference image result in SSIM values closer to 1. For the calculation of SSIM, two images $I$ (reference) and $K$ (reconstructed) of size $m \times n$ are considered. Multiple smaller windows are generated from both images. Consider $x$ and $y$ representing two windows from $I$ and $K$. SSIM is calculated by

24 $$ {\text{SSIM}}\left( {x,y} \right) = \frac{{\left( {2\mu_{x} \mu_{y} + c_{1} } \right)\left( {2\sigma_{xy} + c_{2} } \right)}}{{\left( {\mu_{x}^{2} + \mu_{y}^{2} + c_{1} } \right)\left( {\sigma_{x}^{2} + \sigma_{y}^{2} + c_{2} } \right)}}, $$

where $\mu_{x}$ and $\mu_{y}$ are the mean values over $x$ and $y$ windows. The parameters $\sigma_{x}$, $\sigma_{y}$ and $\sigma_{xy}$ are the variance of $x$ and $y$ and the covariance between $x$ and $y$, respectively. The values $c_{1}$ and $c_{2}$ are constants.
